# Phenotypic characterization of circulating tumor cells in triple negative breast cancer patients

**DOI:** 10.18632/oncotarget.14144

**Published:** 2016-12-24

**Authors:** Sofia Agelaki, Melina Dragolia, Harris Markonanolaki, Saad Alkahtani, Christos Stournaras, Vassilis Georgoulias, Galatea Kallergi

**Affiliations:** ^1^ Laboratory of Translational Oncology, School of Medicine, University of Crete, Voutes, Heraklion, Greece; ^2^ Department of Medical Oncology, University General Hospital of Heraklion, Voutes, Heraklion, Greece; ^3^ Laboratory of Tumor Cell Biology, School of Medicine, University of Crete, Voutes, Heraklion, Greece; ^4^ Department of Zoology, Science College, King Saud University, Riyadh, Saudi Arabia; ^5^ Department of Biochemistry, University of Crete Medical School, Voutes, Heraklion, Greece

**Keywords:** CTCs, HER2, estrogen receptor, progesteron receptor, EGFR

## Abstract

**Introduction:**

Patients with triple negative breast cancer (TNBC), are considered as a poor prognosis group for whom no targeted therapies are currently available. The aim of the present study was to phenotypically characterize their CTCs in order to explore potential therapeutic targets.

**Methods:**

PBMC's cytospins were prepared from 45 early (before and after adjuvant chemotherapy), 10 metastatic TNBC and 21 hormone receptor (HR) -positive patients. The expression of Cytokeratins (CK), ER, PR, EGFR and HER2 on CTCs was assessed using immunofluoresence staining and ARIOL analysis.

**Results:**

In early stage TNBC, ER, PR, HER2 and EGFR expressing-CTCs were detected in 24.4%, 24.4%, 20% and 40% of patients before the initiation of adjuvant chemotherapy, and in 17.8%, 13.3% 6.7% and 51.1% respectively after the completion of adjuvant treatment. Triple staining experiments revealed distinct subpopulations of CTC expressed HR, and ErbB family receptors. In patients with metastatic disease, the frequency of HER2+ CTCs was significantly increased compared to adjuvant setting (60% vs 20%, p=0.014). The presence of CK^+^PR^−^ CTCs, before adjuvant treatment was associated with reduced OS (p=0.032) and DFI (p=0.04). Furthermore, the frequency of ER-, PR- and HER2+ CTCs was higher in HR(+) than in TNBC tumors (57.1%, p=0.006; 52.4%, p=0.021 and 52.38%, p=0.009, respectively).

**Conclusions:**

The CTCs in patients with early TNBC are phenotypically heterogeneous based on the expression of HR, EGFR and HER2 both before and after the completion of adjuvant chemotherapy whereas the presence of HER2^+^ CTCs prevails during disease evolution. These findings could be of clinical relevance in terms of CTC targeting.

## INTRODUCTION

Triple-negative breast cancer (TNBC) represents approximately 15% of all breast cancers, lacks expression of estrogen (ER) or progesterone (PR) receptor, and human epidermal growth factor receptor-2 (HER2) overexpression [1]. Triple negative tumors typically have high grade, elevated mitotic index, and present high levels of tumor cell apoptosis [2]. There is a significant overlap between TNBC and the molecularly defined basal-like intrinsic subtype [3], since up to 80% of basal-like cancers are triple negative and approximately 70% of triple negative tumors are basal-like. EGFR and c-kit have been used as surrogates for the basal-like phenotype [4, 5]. The basal-like phenotype and BRCA1 associated tumors also demonstrate expression of EMT-related molecules such as vimentin and laminin [6, 7]. Chemotherapy remains the standard treatment for TNBC [1, 8]. Although patients with TNBC seem to achieve higher response rates to chemotherapy, this does not translate into superior progression free (PFS) or overall survival (OS) [6]. On the contrary, TNBC is responsible for the highest number of breast cancer-related deaths which is partly attributed to its unique biological characteristics and to the lack of approved targeted treatments for this subtype, highlighting the need for novel therapeutic approaches [9].

Circulating Tumor Cells (CTCs) hold significant prognostic and predictive information in patients with early or metastatic breast cancer [10–12]. Besides the value of CTC detection, the phenotypic and molecular characterization of CTCs can serve as a real time liquid biopsy [13] that can inform on alterations of the tumor's profile which may occur during the evolution of the disease. Several studies have shown a great phenotypic discordance between the primary tumor cells and CTCs especially in HER2 status [14–17]. It has been proposed that the phenotypic analysis of CTCs could reveal therapeutic targets on tumor cells that could be missed when analyzing the primary tumor [15, 18].

It has been recently shown lower CTC positivity rates in TNBC compared to the luminal subtypes possibly attributable to the EMT phenotype of CTCs in these patients [19] although other investigators failed to confirm this observation [20]. Nevertheless, CTC enumeration has been reported to be of prognostic relevance in patients with early stage TNBC [21], as well as in the neoadjuvant [22] and the metastatic setting [23]. However, the phenotypic characterization of CTCs in TNBC patients has not been addressed so far. The aim of the current study was a) to identify the expression pattern of ER, PR, HER2 and EGFR on CTCs of TNBC patients before and after adjuvant chemotherapy b) to delineate the phenotypic heterogeneity of CTCs in patients with early and metastatic TNBC and, finally c) to identify potential differences in the incidence of CTC phenotypes between patients with hormone receptor (HR) positive (+) and TNBC.

## RESULTS

### Expression of ER, PR, HER2 and EGFR on CTCs isolated from early stage TNBC patients

**Figure 1:**
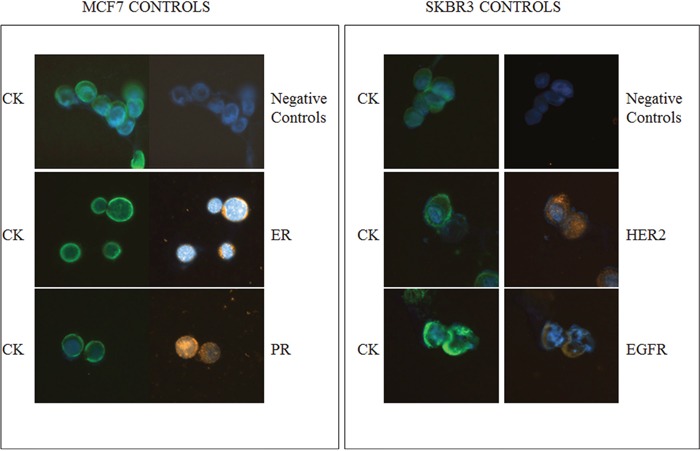
ER, PR, HER2 and EGFR expression in breast cancer cell lines Representative MCF7 cells stained with pancytokeratin (CK) and ER or PR, analyzed by ARIOL microscopy (magnification X400). The SKBR3 cell line was used as positive and negative controls for CK along with HER2 or EGFR, respectively.

**Figure 2:**
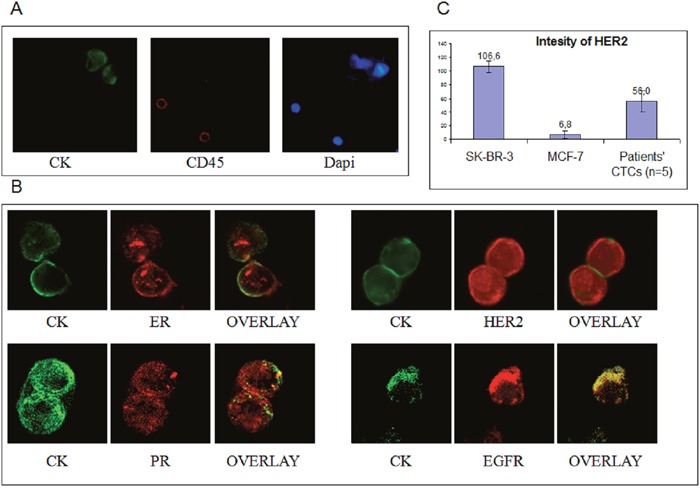
ER, PR HER2 and EGFR staining in TNBC patients **A**. Representative CK+/CD45- CTCs from TNBC patients analyzed by ARIOL microscopy (magnification X400) and Dapi staining; **B**. Representative images from Confocal laser scanning microscopy (magnification X60) of TNBC patients' CTCs stained with CK along with either ER, PR, HER2 or EGFR; **C**. Quantification of HER2 intensity in MCF7, SKBR3 and patients' CTCs.

Cytospins with the corresponding cell lines were used as positive and negative controls in every experiment (Figure 1) The expression of CK was investigated in PBMC s obtained from TNBC patients with early disease before and after the completion of adjuvant chemotherapy, using double CK/CD45 staining experiments (Figure 2A) It was shown that the total number of CK-positive patients, as well as the mean percentage of CK-positive cells per patient, before and after treatment, was not significantly reduced. Indeed, 39 out of 45 (86.6%) patients before and 37 out of 45 (82.2%) patients after the completion of adjuvant treatment were characterized as CTC-positive, since they harvested at least 1 cell positive for at least one of the examined markers No statistical difference was observed in the total number of CTCs between the early and metastatic disease settings (p=0.876).

**Figure 3:**
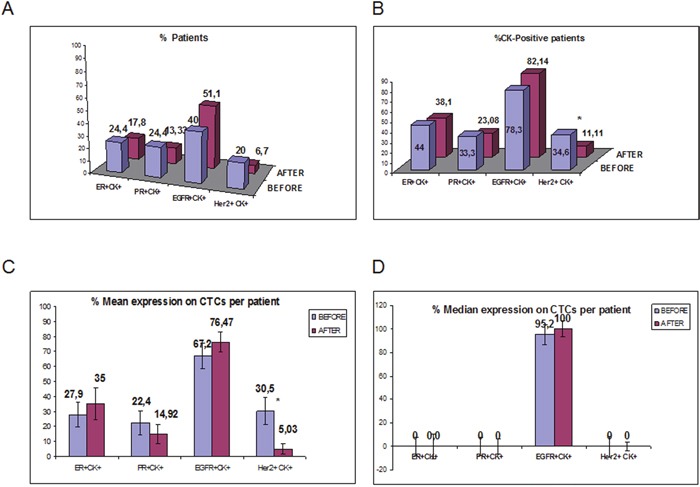
ER, PR, HER2 and EGFR expression on CTCs isolated from TNBC patients before and after adjuvant chemotherapy **A**. Proportion of patients with ER^+^CK^+^, PR^+^CK^+^, HER2^+^CK^+^ and EGFR^+^CK^+^ before and after adjuvant chemotherapy; **B**. Proportion of patients with ER, PR, HER2 and EGFR-positive CTCs, among the CK-positive TNBC patients; **C**. Proportion of CTCs with ER, PR, HER2 and EGFR-expressing CTCs per patient before and after treatment; **D**. % Median expression of ER, PR, HER2 and EGFR-expressing CTCs per patient before and after treatment.

ER-positive CTCs (Figure 2B) were identified in 11 out of 45 patients (24%) before and in 8 (17.8%) after chemotherapy (Figure 3A). Among CTC-positive patients, the incidence of ER-positive CTCs before and after chemotherapy was 44% and 38.1% respectively (Figure 3B). The mean percentage of ER positive CTCs per CK-positive patient was 27.9% before and 35% after treatment (p=0.334; Figure 3C).

Similar positivity rates were observed for PR-positive CTCs (Figure 2B) [24.4% (11 out of 45 patients) and 13.3% (6 out of 45), before and after chemotherapy, respectively] (Figure 3A); furthermore, among patients with detectable CTCs, the corresponding numbers were 33.3 % and 23.1%, respectively (Figure 3B). The mean percentage of CTCs expressing PR per CK-positive patient was 22.4% before and 14.9% after treatment (p=0.466; Figure 3C). There was a significant correlation between the proportion of ER- and PR-positive CTCs (p=0.016) before the initiation of adjuvant treatment.

The frequency of HER2-positive CTCs (Figure 2B) was decreased (p=0.05) after treatment [in 9 out of 45 patients (20%) pre-, and in 3 out of 45 patients (6.7%) post-adjuvant chemotherapy] (Figure 3A). Among CTC-positive patients, the corresponding percentages were 34.6% and 11.1%, before and after chemotherapy, respectively (Figure 3B). Furthermore, the mean percentage of HER2-expressing CTCs/patient was also significantly reduced (30.4% at baseline and 5.0% after adjuvant chemotherapy; p=0.011) (Figure 3C) while the number of HER2-/CK+ CTCs was increased after the completion of adjuvant therapy from 69.55% to 94.97% (p=0.013). The median expression is also shown in Figure 3D. The quantification of HER2 expression in MCF7 SKBR3 and patients' CTCs (n=5 patients) is shown in Figure 2C

.

EGFR-expressing CTCs were identified in 18 out of 45 patients (40%) before the initiation of adjuvant chemotherapy and in 23 out of 45 patients (51.1%) after treatment (Figure 3A). Among the patients with detactable CTCs, EGFR expression before and after chemotherapy was observed in 78.3% and 82.1%, respectively (Figure 3B). The mean percentage of EGFR-expressing CTCs was 67.2% before and 76.5% after treatment (p=0.442; Figure 3C). The median expression per patient for EGFR-positive CTCs was high both before (95.2%) and after (100%) treatment (Figure 3D). The number of CTCs isolated from TNBC patients for each phenotype is shown in Table 1.

**Table 1:** CTCs' phenotype in TNBC patients before and after adjuvant treatment

EARLY TNBC PATIENTS																
PATIENTS	CTCs' PHENOTYPE BEFORE CHEMOTHERAPY								CTCs' PHENOTYPE AFTER CHEMOTHERAPY							
	ER^+^	ER^−^	PR^+^	PR^−^	EGFR^+^	EGFR^−^	Her2^+^	Her2^−^	ER^+^	ER^−^	PR^+^	PR^−^	EGFR^+^	EGFR^−^	Her2^+^	Her2^−^
1	1	21	0	1	0	1	1	0	1	3	0	1	1	0	0	0
2	1	1	1	2	40	2	1	3	0	2	0	3	0	0	0	0
3	0	0	0	2	2	0	1	0	0	1	0	1	0	0	0	0
4	0	0	0	0	0	0	0	0	1	0	0	0	2	1	0	0
5	1	0	5	5	1	0	0	6	0	2	0	3	1	1	2	14
6	0	4	1	2	0	0	1	1	0	0	0	0	0	0	0	0
7	0	0	0	0	0	0	0	0	NS	NS	NS	NS	NS	NS	NS	NS
8	0	0	0	0	0	0	0	0	0	0	1	3	2	0	0	1
9	0	3	0	3	5	0	0	3	0	3	0	3	2	0	0	3
10	0	1	0	3	0	0	0	0	1	0	0	1	0	0	0	5
11	0	0	0	2	0	0	0	0	0	0	0	3	0	0	0	3
12	0	0	0	7	0	0	0	0	1	0	0	0	2	2	0	3
13	1	0	0	1	6	1	18	0	0	1	0	6	1	1	0	0
14	0	1	0	2	0	0	0	0	0	0	0	6	2	0	0	3
15	1	5	4	0	1	1	0	1	0	0	0	0	1	1	0	0
16	0	0	0	0	1	1	0	7	0	0	1	0	0	0	0	2
17	0	1	0	1	1	0	0	2	NS	NS	NS	NS	NS	NS	NS	NS
18	0	0	0	2	1	0	0	0	NS	NS	NS	NS	NS	NS	NS	NS
19	0	0	1	7	0	0	1	0	0	0	0	0	3	0	0	1
20	0	1	0	4	1	0	0	0	0	0	0	0	0	0	0	0
21	0	0	0	0	0	0	0	0	0	0	0	0	0	0	0	2
22	0	0	0	0	1	0	0	2	1	0	0	2	3	0	0	4
23	0	2	0	75	0	2	0	1	0	1	0	1	0	0	0	1
24	1	1	0	1	0	0	0	3	1	0	3	4	1	0	0	0
25	0	1	0	1	2	0	**0**	**1**	0	0	0	3	0	1	0	1
26	0	0	1	0	0	0	0	0	0	0	0	1	1	0	0	1
27	0	12	0	62	7	0	15	0	0	6	0	4	2	0	4	1
28	0	0	0	8	0	0	0	0	0	0	0	5	3	0	0	2
29	0	0	0	1	0	0	0	1	0	0	0	6	2	0	0	5
30	0	0	0	2	0	0	0	0	0	0	0	0	1	3	0	0
31	23	18	9	1	0	3	0	1	0	0	0	0	7	3	1	2
32	0	0	0	1	0	1	0	1	1	0	1	0	0	0	0	1
33	0	4	2	1	0	0	0	0	0	0	0	4	5	0	0	1
34	0	0	0	2	0	0	0	2	0	25	0	10	1	1	0	8
35	0	0	0	0	0	0	0	0	0	0	2	2	1	0	0	1
36	2	6	0	5	4	6	3	0	0	3	0	0	0	0	0	1
37	0	1	2	0	0	0	0	2	NS	NS	NS	NS	NS	NS	NS	NS
38	1	0	1	0	0	0	0	1	0	0	0	1	0	0	0	0
39	0	0	0	0	0	0	0	0	0	0	0	2	0	0	0	4
40	0	1	0	0	1	0	0	0	0	3	1	0	6	0	0	3
41	16	1	0	2	3	1	0	1	0	3	0	4	0	1	0	0
42	0	0	0	0	0	0	0	0	NS	NS	NS	NS	NS	NS	NS	NS
43	0	3	0	0	1	0	0	0	0	0	0	0	0	0	0	0
44	1	0	14	5	0	1	1	0	0	1	0	0	1	0	0	0
45	0	1	0	1	1	1	0	1	3	1	0	1	0	0	0	0

**Figure 4:**
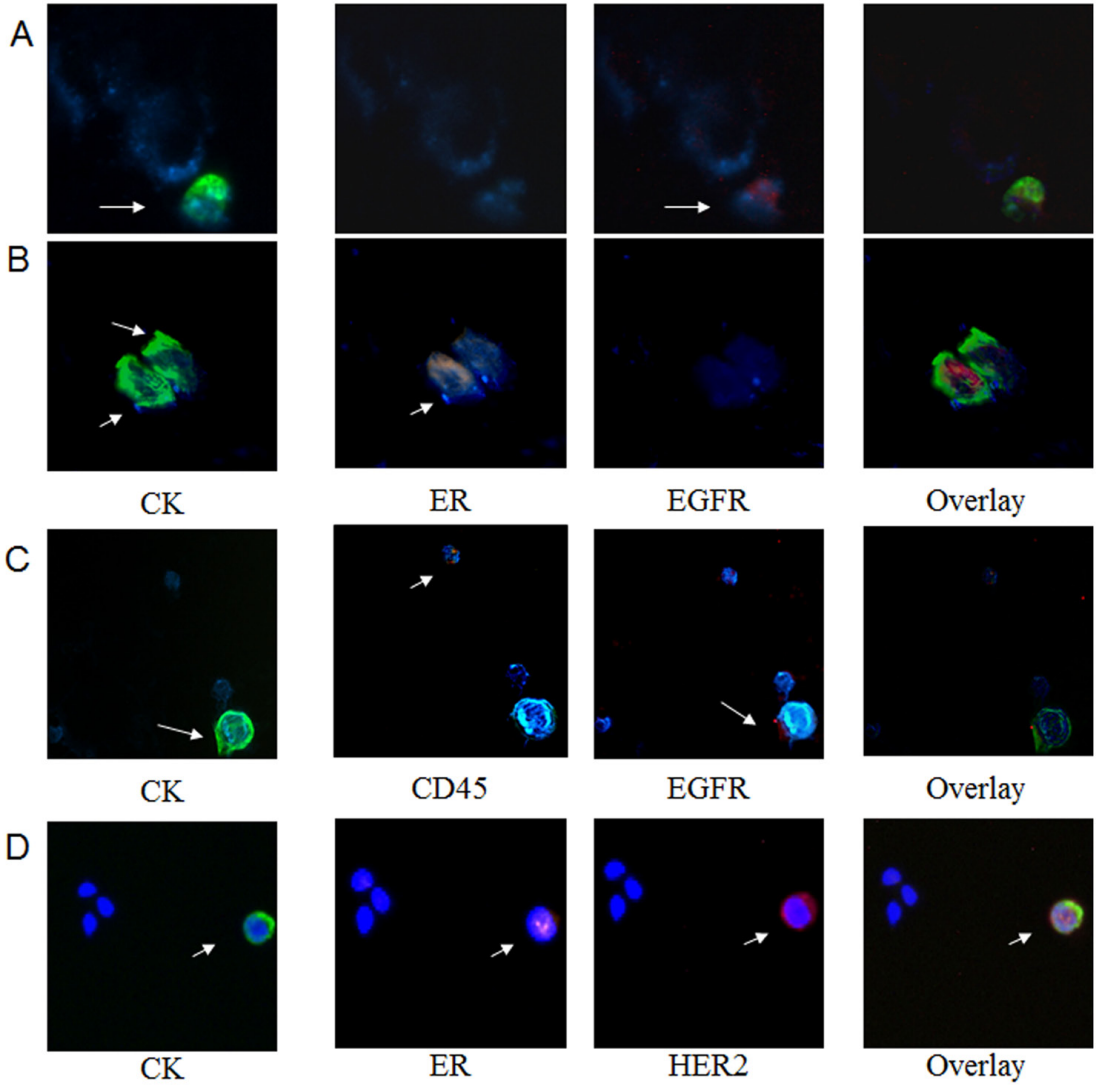
Phenotypic characterization of CTCs after Immumomagnetic separation Representative triple (CK/ER/EGFR and CK/ER/HER2) immunofluorescence staining of CTCs isolated from early stage TNBC patients using ARIOL microscopy (magnification X400). **A**. CTC positive for CK/EGFR and negative for ER; **B**. CTC positive for CK/ER and negative for EGFR; **C**. CTC positive for CK, EGFR and negative for CD45; **D**. CTC positive for CK/HER2/ER.

To identify the potential co-expression of hormone receptors with ErbB family receptors at the single cell level, triple staining experiments for CK/ER/EGFR, CK/ER/HER2, CK/PR/EGFR and CK/PR/HER2) were performed after negative immunomagnetic isolation of CTCs. No co-expression was observed for EGFR and HR as it is shown in Figure 4A and Figure 4B where CK/ER/EGFR staining revealed that distinct subpopulations were identified in individual patients. On the other hand, in 3 patients, HER2 was co-expressed with ER (Figure 3D), whereas HER2 and PR co-expression was not evident. In addition in order to reassure that the cells characterized as CTCs after immunomagnetic isolation are CD45-negative we performed triple staining experiments with anti-CK/CD45/EGFR antibodies (Figure 4C).

### Phenotypic characterization of CTCs in patients with metastatic TNBC

**Figure 5:**
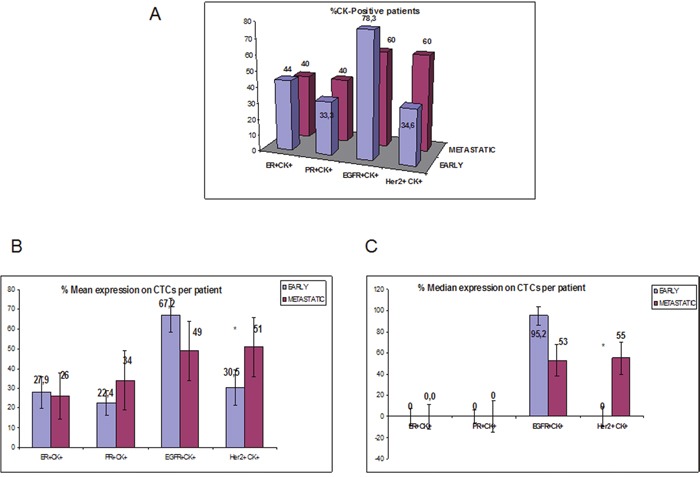
ER, PR, HER2 and EGFR expression on CTCs isolated from early vs metastatic TNBC patients **A**. Percentage of patients that expressed double positive cells of each phenotype (ER^+^CK^+^, PR^+^CK^+^, HER2^+^CK^+^ and EGFR^+^CK^+^) in early vs metastatic setting; **B**. % Mean expression of ER, PR, HER2 and EGFR-expressing CTCs per patient in early vs metastatic setting; **C**. % Median expression of ER, PR, HER2 and EGFR-expressing CTCs per patient in early vs metastatic setting.

To identify potential differences in CTC phenotype between early and metastatic TNBC patients, double staining experiments were also performed in 10 CTC-positive patients with metastatic disease before the initiation of any systemic treatment. ER-positive CTCs were observed in 4 (40%) out of 10 patients compared to 44% in patients with early stage disease. Similarly, the percentage of ER-negative CTCs per patient was not significantly different in metastatic compared to early patients (72% vs 74%; p=0.082) (Figure 5A).

PR-expressing CTCs were identified in 4 out of 10 (40%) metastatic and in 33.3% patients with early breast cancer. The percentage of PR-positive CTCs per patient was not statistically different between the two settings (34% vs 22.3% respectively) (p= 0.231; Figure 5B).

HER2-expressing CTCs were detected in 6 out of 10 (60%) patients with metastatic disease and in 9 out of 26 (34.6%) with early stage disease. Furthermore, there was a significantly increased number of HER2-expressing CTCs in patients with metastatic compared to patients with early stage disease (51% vs 30.5%, respectively p=0.014; Figure 5B).

EGFR-positive CTCs were also detected in 60% and 78.3% of patients with metastatic and early stage disease, respectively (Figure 5A). The percentage of EGFR-positive CTCs was high but not significantly different (p=0.337; Figure 5B) in both settings.

The median expression of ER, PR, HER2 and EGFR-expressing CTCs per patient as well as the number of the corresponding CTCs in the metastatic setting are presented in Figure 5C and Table 2, respectively.

**Table 2:** CTCs' phenotype in metastatic TNBC patients

CTCs' PHENOTYPE IN METASTATIC TNBC PATIENTS								
PATIENTS	ER^+^	ER^−^	PR^+^	PR^−^	EGFR^+^	EGFR^−^	Her2^+^	Her2^−^
1	0	1	0	3	1	0	1	0
2	1	0	1	0	4	1	2	2
3	2	4	3	4	0	0	1	0
4	0	13	0	2	0	1	3	0
5	0	1	0	1	3	8	0	0
6	1	1	0	17	1	0	0	1
7	0	2	0	1	7	1	0	1
8	0	1	1	0	1	0	4	2
9	0	1	0	1	0	1	0	1
10	3	1	9	0	0	0	2	0

### ER, PR, HER2 and EGFR expression on CTCs from patients with HR(+) breast cancer

**Figure 6:**
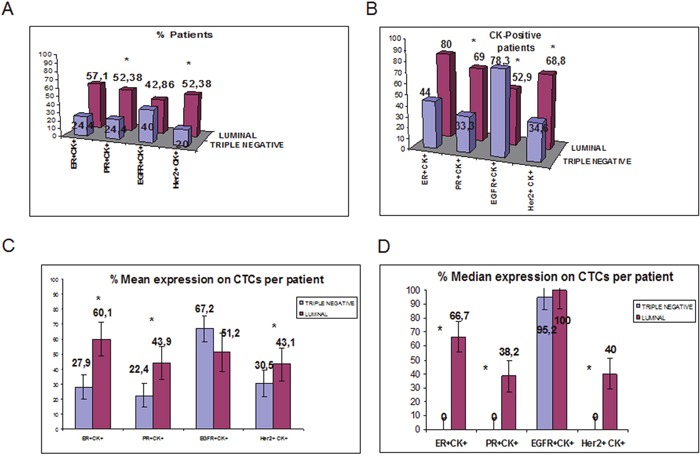
Comparison of ER-, PR-, HER2- and EGFR-expressing CTCs detected in TN and HR-positive breast cancer patients before adjuvant chemotherapy **A**. Proportion of patients with ER^+^CK^+^, PR^+^CK^+^, HER2^+^CK^+^ and EGFR^+^CK^+^ in TN and HR-positive patients; **B**. Proportion of patients with ER, PR, HER2 and EGFR-positive CTCs, among the CK-positive TN and HR-positive patients; **C**. Proportion of CTCs with ER, PR, HER2 and EGFR-expression inTNBC and HR-positive patients; **D**. Median expression of ER, PR, HER2 and EGFR-expressing CTCs per TNBC and HR-positive patients

The expression of the above markers was also assessed on CTCs isolated from 21 patients with HR(+) early breast cancer before the initiation of adjuvant treatment. ER-expressing CTCs were detected in 12 out of 21 patients (57.1%) with HR(+) tumors compared to TNBC patients (24.4%; (p=0.232) (Figure 6A). Among the CK-positive patients, ER-positive CTCs were identified in 12 out of 15 patients (80%) while the respective percentage for TNBC was 44% (p=0.313; Figure 6B). The mean percentage of ER-expressing CTCs per patient was significantly higher in HR (+) patients compared to TNBC patients [60.1% versus 27.9%, respectively] (p=0.006; Figure 6C).

Similarly, PR-expressing CTCs were identified in 11 out of 21 HR(+) patients (52.4%) compared to 24.4% in TNBC (p=0.036; Figure 6A). Among the CK-positive patients, PR was expressed in 69% [11 out of 16 patients] with HR(+) tumors vs 33.3% in patients with TNBC (p=0.016; Figure 6B). The percentage of PR-expressing CTCs was 43.9% in patients with HR(+) disease and 22.4% in TNBC patients (p=0.028; Figure 6C).

HER2-expressing CTCs were detected in 11 out of 21 patients (52.4%) with HR(+) tumors compared to 20% of TNBC patients (p=0.023). Among the patients with detectable CTCs the respective numbers were 68.8% (11 out of 16 patients) in HR(+) and 34.6% in TNBC patients (p=0.055; Figure 5B). Moreover, the percentage of CTCs expressing HER2 was also significantly higher (p=0.029) in patients with HR(+) tumors compared to patients with TNBC tumors(43.1% vs 30.5%) (Figure 5C).

In the whole cohort of patients, EGFR-expressing CTCs were detected in 42.9% (9 out of 21) vs 40% in HR+ and TNBC subjects (p= 0.785) respectively (Figure 5A). However, among the CK-positive patients, EGFR-expressing CTCs were detected in 9 out of 17 patients (52.9%) with HR(+)- disease compared to 78.3% of TNBC patients (p=0.037) (Figure 5B). The percentage of CTCs expressing EGFR per patient was 51.2% versus 67.2% in the two different groups of patients (p=0.47; Figure 5C).

The median expression of each examined molecule per TNBC and HR-positive patient is shown in Figure 5C.

### CTC phenotype and clinical outcome

After a median follow-up period of 55 months (range, 16- 88 months), 10 (21.3%) patients with early TNBC presented disease relapse. Seven out of 21 (33.3%) of patients with CK^+^ER- CTCs relapsed; the incidence of relapse was significantly increased (p=0.048) in patients with CK+ER- CTCs at baseline compared to patients without this CTC subpopulation before the initiation of adjuvant chemotherapy. Similarly, seven out of 19 patients (36.8%) with CTCs bearing the CK+HER2- phenotype at baseline relapsed during the follow-up period compared to 2 out of 26 (7.6%) patients who had not this phenotype (p=0.044). Cox regression analysis revealed shorter DFI (p=0.04; HR: 1.035; Cl: 1.002-1.070) for patients harvesting CK+PR- CTCs in their blood. There was no correlation between DFI and the other CTC subpopulations (data not shown).

Similarly, only the CK+PR- phenotype before adjuvant treatment was associated with a reduced OS (p=0.034; HR:1.04; CI: 1.003-1.075. Table 3); it should be mentioned that, during the follow up period, there were no deaths among patients lacking this phenotype.

**Table 3:** Survival analysis in TNBC patients before and after chemotherapy

CTCs' PHENOTYPE	BEFORE CHEMOTHERAPY			AFTER CHEMTHERAPY				BEFORE CHEMOTHERAPY		AFTER CHEMTHERAPY		
			OS						DFS			
	*p* values	*Median OS (range)*	HR	*p* values	*Median OS (range)*	HR	*p* values	*Median OS (range)*	HR	*p* values	*Median OS (range)*	HR
**ER+CK+**	0,705	76,2 (76,6-94,5)	0,854	0,721	64,3 (46,1-82,54)	1,21	0,52	74,25 (58,9-89,6)	0,892	0,556	64,3 (46,1-82,5)	0,716
**ER-CK+**	0,523	73,5 (61,3-85,7)	0,878	0,657	67,5 (54,9-80,2)	0,912	0,534	89,6 (48,4-130,9)	0,955	0,582	60,4 (47,8-73)	1,024
**PR+CK+**	0,495	82 (71,8-92,1)	0,588	0,127	54 (37-70,9)	2,018	0,517	80 (61,9-98,1)	0,87	0,39	54 (37-70)	1,481
**PR-CK+**	**0,034**	55 (16-94)	1,038	0,816	53,5 (16-80)	0,957	**0.04**	66 (42,8-133,2)	1,035	0,627	61,2 (51,1-71,2)	1,054
**HER2+CK+**	0,647	74,6 (61,5-97,7)	0,887	0,682	54,5 (52-57)	0,265	0,546	62,071 (52,2-71,9)	0,921	0,484	56 (55-57)	0,182
**HER2-CK+**	0,331	74,8 (61,7-87,9)	1,239	0,707	73,3 (65,9-80,8)	0,923	0,255	90,1 (47,9-132,1)	0,218	0,698	58,7 (50,1-67,4)	1,039
**EGFR+CK+**	0,145	79,7 (65,5-93,9)	1,044	0,157	84,4 (77,7-91,2)	0,253	0,254	101,4 (61,1-141,8)	1,031	0,127	76,4 (63,1-89,7)	0,439
**EGFR-CK+**	0,534	76,5 (61,9-91,1)	1,196	0,362	61 (39-67)	0,06	0,232	74,5 (58,1-90,9)	1,248	0,2	77,7 (62,7-92,6)	0,401

## DISCUSSION

Treatment of patients with TNBC remains challenging due to the absence of effective targeted therapies that could improve clinical outcome. However, it is widely accepted that TNBC are often characterized by an extremely high heterogeneity [24]. Since it is difficult to recognize the “hazardous” subpopulation that are the origin of metastases in breast cancer, it was of interest to extensively characterize the CTCs of TNBC patients. The present study phenotypically characterized the CTCs in TNBC patients and, more especially, evaluated the expression of ER, PR, HER2 and EGFR on these cells. The presented data clearly indicate that ER and PR were expressed on CTCs detected in 24.4% of patients with early stage disease.. Similarly, CK+HER2+ CTCs could be detected in 20% of TNBC patients with early stage disease; conversely, CK+EGFR+ CTCs (40%) were frequently detected in these patients and clearly predominated over the other phenotypes. This observation seems to indicate that in TNBC patients the phenotype of CTCs reflects the well known expression of EGFR in the primary tumor cells [25].

The presence of ER+CK+ at baseline correlated significantly with the presence of PR+CK+ (p=0.016), implying that these phenotypes are frequently observed in the same patient. Conversely, there was no statistical correlation between the expression of ER or PR and EGFR on CTCs, suggesting that these molecules are expressed in different subpopulations. This notion was further confirmed by the absence of co-expression of ER or PR and EGFR in triple staining experiments.

An interesting observation in the current study was the increased incidence of disease relapse in patients with CK+ER- CTCs; this finding strongly suggests that the absence of HR expression in CTCs is a poor prognostic factor in TNBC patients. Moreover, survival analysis revealed that patients with CTCs bearing the CK+PR- phenotype before treatment experienced a significantly decreased DFI (p=0.04) and OS (p=0.032) suggesting that these cells could be more aggressive in terms of metastatic potential. These observations seem to be in agreement with the worse clinical outcome of patients with HR-negative tumors [26–29]. However, these results are only exploratory, retrospectively performed and need to be confirmed in a prospective study including a larger group of patients. In addition, we have to mention that there is a possibility to underestimate the total number of CTCs because of the low CK8/18 expression in TNBC patients.

In the present study it was also of interest to investigate the different subpopulations of CTCs surviving adjuvant chemotherapy. Interestingly, chemotherapy resulted in a reduced frequency of HER2- but not of ER- or PR-expressing CTCs. This observation suggests that chemotherapy cannot eliminate the different subpopulations of CTCs with the same efficacy. This assumption strongly suggests the need for additional therapeutic approaches in order to eliminate these chemotherapy-resistant CTCs. In line with this observation, it has been shown that CTCs could have EMT and stem cell properties [30–33]. These phenotypes are potentially resistant to common chemotherapy as it has been recently reported in patients receiving neoadjuvant treatment t [34].

It is also noteworthy that the incidence of CK+HER2+-expressing CTCs in patients with metastatic TNBC was significantly increased compared to patients with early stage disease; this finding clearly suggests that CK+HER2+ is an aggressive phenotype that predominates during disease evolution even in TNBC patients. The potential to target CK+HER2+ CTCs with lapatinib or trastuzumab, has been recently reported [35, 36]. These observations suggest that therapies directed against specific targets could be necessary in order to eliminate CK+HER2+ chemotherapy-resistant CTCs.

Finally, the comparative distribution of the evaluated CTC subpopulations in HR(+) and in TNBC patients has shown that ER- and PR-positive phenotypes are significantly increased in HR-positive breast cancer patients.. Moreover, HER2 expression on CTCs was higher in HR(+) compared to TNBC patients, confirming the observation that HER2 can be present on CTCs irrespectively of the phenotype of the primary tumor [14, 36].

## MATERIALS AND METHODS

### Patient samples and cytospin preparation

A total of 76 CTC-positive patients with early or metastatic breast cancer were included in the present study; 55 patients had TNBC (n=45 with early and n=10 with metastatic disease) and 21 had HR(+) early breast cancer. Patients' characteristics are shown in Table 4. These patients had been identified after routine screening performed for the presence of CK(+)/CD45(−) cells on PMBC cytospins. Ten female normal blood donors were also included as negative controls. Peripheral blood (10 ml in EDTA) was obtained beforethe initiation of adjuvant (usually within 3-4 weeks after primary surgery) or first line therapy for metastatic disease. TNBC patients with early disease were also evaluated at the end of adjuvant chemotherapy. All blood samples were obtained in the middle of vein puncture after the first 5 ml of blood were discarded. These precautions were undertaken in order to avoid contamination of the blood sample with epithelial cells from the skin during sample collection. All patients gave their informed consent to participate in the study, which has been approved by the Ethics and Scientific Committees of our Institution.

**Table 4:** Patients' characteristics

Early disease (45 TNBC patients)		Early disease (21HR-positive)		Metastatic TNBC patients (10 Patients)	
**Age**		**Age**		**Age**	
Median, range	53 (35-77)	Median, range	57 (37-84)	Median, range	66 (45-82)
	*N*		*N*		*N*
**Menopausal status**		**Menopausal status**		**Menopausal status**	
Premenopausal	11 (24.4%)	Premenopausal	5 (23.8%)	Premenopausal	2(20%)
Postmenopausal	28(62.2%)	Postmenopausal	12(57.1%)	Postmenopausal	7(70%)
Unknown	6(13.3%)	Unknown	4(19%)	Unknown	1(10%)
**Tumor size**		**Tumor size**		**Disease sites**	
pT1	15(33.3%)	pT1	2(9.5%)	1	6(60%)
pT2	19(42.2%)	pT2	6(28.6%)	2	1(10%)
pT3	1(2.2%)	pT3	6(28.6%)	3	1(10%)
Unknown	10(22%)	Unknown	8(38%)	≥4	1(10%)
				Unknown	1(10%)
**Lymph node status**		**Lymph node status**		**Predominantly visceral disease**	
Node-negative	23(51.1%)	Node-negative	3 (14.3%)	Yes	2 (20%)
Node-positive	12(26.7%)	Node-positive	12(57.1%)	No	8(80%)
Unknown	10(22%)	Unknown	6(28.6%)	Unknown	0(0%)
**Histologic grade**		**Histologic grade**		**Primary breast cancer**	
Grade 1	1(2.2%)	Grade 1	0(0%)	Adjuvant	3(30%)
Grade 2	8(17.8%)	Grade 2	6(28.6%)	Metastatic	6(60%)
Grade 3	27(60%)	Grade 3	9(42.9%)	Unknown	1(10%)
Grade 4	2(4.4%)	Grade 4	1(4.8%)		
Unknown	7(15.5%)	Unknown	5(23.8%)		
**ER/PR tumor status**		**ER/PR tumor status**		**ER/PR tumor status**	
Positive	0(0%)	Positive	21(100%)	Positive	0(0%)
Negative	45(100%)	Negative	0(0%)	Negative	10(100%)
Unknown	0 (0%)	Unknown	0 (0%)	Unknown	0 (0%)
**HER2 tumor status**		**HER2 tumor status**		**HER2 tumor status**	
Positive*	0(0%)	Positive*	0(0%)	Positive*	0(0%)
Negative	45(100%)	Negative	21(100%)	Negative	10(100%)
Unknown	0(0%)	Unknown	0(0%)	Unknown	0(0%)

Peripheral blood mononuclear cells (PBMC) were isolated with Ficoll-Hypaque density gradient (d=1, 077gr/mol) centrifugation at 1800rpm for 30min. PBMCs were washed three times with PBS and centrifuged at 1500rpm for 10min. Aliquots of 250.000 cells were centrifuged at 2000rpm for 2min on glass slides. Cytospins were dried up and stored at −80°C. Four to five slides were evaluated for each patient.

### Cell cultures

For control experiments two different breast cancer cell lines, MCF7 and SKBR3 (both obtained from the ATCC; American Type Culture Collection, USA), were used. The MCF7 mammary adenocarcinoma cells were cultured in (v/v) 1:1 Dulbecco's Modified Eagle Medium (DMEM)/Ham's F12 medium (GIBCO-BRL Co, MD, USA) supplemented with 10% fetal bovine serum (FBS) (GIBCO-BRL), 2mM L-glutamine (GIBCO-BRL) 30mM NaHCOB_3B_, 16 ng/ml insulin and 50 mg/ml penicilline/streptomycin (GIBCO-BRL). SKBR3 cells were used as positive control for HER2 and EGFR expression. SKBR3 cells were cultured in McCoy's (GIBCO-BRL) enriched with 10% fetal bovine serum and 2mM L-glutamine supplemented with 50 mg/ml penicilline/streptomycin. Cells were maintained in a humidified atmosphere of 5% COB_2B_-95% air.

Sub-cultivation for all cell lines was performed with 0.25% trypsin and 5mM EDTA. All experiments were performed during the logarithmic growth phase. 15-20 h before each experiment and cells were transferred to serum-starved medium containing only L-glutamine, NaHCOB_3B_ and antibiotics.

### Confocal laser scanning and ARIOL system Microscopy

The A45-B/B3 mouse antibody (Micromet Munich, Germany) which detect the CK8, CK18 and CK19 was used in order to evaluate the expression of cytokeratins on PBMCs cytospins; The A45-B/B3 antibody is commonly used for CTCs evaluation [17] in breast cancer, however we have to mention that TN tumors frequently express lower level of 8/18 [37]. Cytospins were also double stained with anti-CD45 (common leukocyte antigen) antibody to exclude possible ectopic expression of cytokeratins by hematopoietic cells. In cytospins of the same patients the expression of ER (Santa Cruz, Santa Cruz, CA, USA), PR (Santa Cruz), HER2 (Cell Signaling, Boston, US) and EGFR (Santa Cruz), was also evaluated using double staining experiments. The antibodies against the ER and PR were appropriate for nuclear staining according to the manufacturer.

PBMC cytospins were fixed with cold aceton:methanol 9:1 for 20min and stained for cytokeratin with a pancytokeratin antibody as mentioned above. Subsequently, the same slide was stained with ER, PR, HER2 or EGFR antibodies for 1h. Cells were then incubated with the corresponding secondary antibodies for 45 min. Slides were analyzed using either confocal laser scanning microscopy (Leica Lasertechnik, Heidelberg, Germany) or the semi-automated ARIOL system [16, 17].

Positive and negative controls were included in each experiment. Negative controls were prepared by omitting the corresponding primary antibodies. Furthermore, MCF7 breast cancer cells were used as negative controls for HER2 and EGFR expression and SKBR3 cells served as negative controls for ER and PR expression. The cyto-morphological criteria proposed by Meng et al [38] were employed in order to characterize a CK-positive cell as a CTC. All the CTCs displaying a HER2 staining intensity higher than that of MCF7 and PBMCs, were considered as HER2-positive as it has been previously reported [16, 17]. The intensity of HER2 was also quantified in MCF7, SKBR3 and in CTCs isolated from 5 TNBC patients using ARIOL system analysis. For ER and PR only the nuclear staining was accepted as positive irrespectively of the intensity. Finally, for EGFR, intensity higher than that of PBMCs and MCF7 (negative controls) was considered as positive.

### Immunomagnetic separation of CTCs

In patients with detectable CTCs expressing at least 2 among the ER, PR, HER2 or EGFR molecules, a negative immunomagnetic selection for CTC isolation was performed according to Naume et al (1998) in a separate blood sample. Briefly, 100μl of CELLection beads coated with anti-CD45 monoclonal antibody (Dynal, Invitrogen, Carlsbad, CA, USA) were added in 10^7P^/ml PBMCs in PBS/0.1% BSA/2mM EDTA. PBMCs were isolated from 20 ml of blood using Ficoll density gradient centrifugation. After incubation for 30min at 4°C, the supernatant was transferred in FBS-coated tubes and cells were cyto-centrifuged at 2000rpm for 2min on glass slides.

### Triple immunofluorescence

Triple immunofluoresence staining experiments for CK/ER/HER2, CK/ER/EGFR, CK/PR/HER2 and CK/PR/EGFR were performed on PBMC cytospins prepared after immunomagnetic separation. Cells were initially fixed with cold aceton:methanol 9:1. After blocking with PBS supplemented with 10% (v/v) FBS for 30min, cells were incubated with the corresponding antibodies for 45min each. Zenon technology (FITC-conjugated IGg1 antibody) (Molecular Probes, Invitrogen, Carlsbad, CA, USA) was used for CK detection with the A45-B/B3 antibody. Zenon antibodies were prepared within 30min before use.

HER2 (Oncogene, Dermstadt, Germany) was detected using anti-mouse antibody labelled with Alexa 555 (Molecular Probes), whereas the ER and PR were detected with the previously described anti-rabbit antibodies and Alexa 633 fluorochrome (Molecular Probes). EGFR was detected with anti-mouse (Santa Cruz) antibody and Alexa 555 fluorochrome. Finally, cells were stained with DAPI conjugated with antifade.

### Statistical analysis

Disease-Free Interval (DFI), was defined as the time from the initiation of adjuvant treatment until the day of the first evidence of disease recurrence or death from any cause and overall survival (OS) as the time from disease diagnosis to death from any cause. Progression-Free Survival (PFS) in patients with metastatic disease was defined from the initiation of front-line treatment until disease relapse or death. Kaplan-Meier curves and Cox regression analysis for PFS and OS were compared using the log-rank test to provide a univariate assessment of the prognostic value of selected clinical risk factors. Clinico-pathologic factors such as menopausal status, tumor size, number of involved lymph nodes, estrogen and progesterone receptor (ER) status and HER-2 status were also evaluated in univariate analysis. Variables that were found to be significant at the univariate analysis were then entered in a stepwise multivariate Cox proportional hazards regression model to identify those with independent prognostic information. All statistical tests were performed at the 5% level of significance. SPSS version 15 (SPSS Inc, Chicago, IL) statistical software was used for the analysis.

## CONCLUSIONS

CTCs isolated isolated from TNBC patients express ER, PR and HER2 although the freaquency is significantly lower compared to those detected in patients with HR-positive tumors. The incidence of CK+HER2+ CTCs is increased in metastatic TNBC patients suggesting that this phenotype prevails during disease evolution. The CK+EGFR+ CTC phenotype was the most frequently detected among TNBC patients both before and after the completion of adjuvant chemotherapy; subsequent studies could elucidate whether EGFR could represent an attractive therapeutic target in patients with metastatic TNBC.
